# Birth Weight Stratification May Clarify Developmental Effects of Parent‐Delivered Interventions in Preterm Infants: Letter to the Editor

**DOI:** 10.1002/hsr2.72489

**Published:** 2026-05-04

**Authors:** Yudai Kaneda

**Affiliations:** ^1^ Clinical Training Center Jyoban Hospital of Tokiwa Foundation Fukushima Japan; ^2^ London School of Hygiene & Tropical Medicine London UK

I read with great interest the randomized trial by Monfared et al. examining the effect of maternal massage with verbal contact on attachment and motor performance in preterm infants [1]. The authors should be commended for addressing parent‐delivered developmental support; however, I contend that an important and clinically well‐established stratification variable, birth weight category, is not sufficiently considered in the analysis or discussion.

A large body of perinatal research supports the use of birth weight categories (e.g., very low birth weight [VLBW, < 1500 g] and low birth weight [1500–2499 g]) as predictors of neurodevelopmental and health outcomes, sometimes more consistently than gestational age alone; for instance, in extremely preterm infants, birthweight models yielded an area under the curve (AUC) of 0.80 for neurodevelopmental impairment/death risk, comparable or superior to gestational age (AUC 0.79) [2]. Birth weight stratification has long been a standard framework for reporting neonatal outcomes in NICU graduate follow‐up programs, and epidemiological reviews further demonstrate that neurodevelopmental impairments and behavioral disorders decrease as both gestational age and birth weight increase, with greater GA at birth and higher birth weight directly associated with a lower risk of developmental delay across language, cognitive, sensory, and motor domains, suggesting an interaction between physical immaturity and developmental risk [3, 4]. Importantly, while much of the literature focuses on VLBW infants, preterm children even in the 1500–1999 g range have been reported to experience “considerable delays in several developmental domains,” particularly social (46%; 95% confidence interval 36.6%–55.7%), general adaptive (26%; 18.4%–35.4%), conceptual (17%), and practical (21%) domains on Bayley‐III scales at 18–24 months corrected age, indicating meaningful heterogeneity within the broader low‐birth‐weight population [5].

In the current study, the authors report no statistically significant differences in baseline birth weight between the intervention group (mean ≈1868.6 ± 416 g) and the control group (≈1870.0 ± 388 g), with similarly balanced demographic characteristics overall. However, such aggregate comparisons may obscure heterogeneous risk profiles within a cohort defined only by a < 2500 g threshold. A lack of difference in mean birth weight does not preclude the presence of effect modification, whereby infants in the < 1500 g stratum may respond differently to sensory‐motor and attachment‐focused interventions than those weighing 2000–2500 g, owing to differences in physiological maturity, sensory processing, and neurodevelopmental reserve.

Given that detailed baseline birth weight data are already reported in the present study, it may be feasible for Monfared et al. to further explore whether the effects of maternal massage with verbal contact differ across birth weight categories (e.g., < 1500 g, 1500–2000 g, and 2000–2500 g). Such stratified analyses could help clarify whether this intervention is particularly beneficial for specific subgroups of preterm infants, or whether responsiveness varies according to physiological maturity and neurodevelopmental reserve associated with birth weight. If additional analyses are possible, presenting these results would provide highly valuable insights for clinicians involved in NICU follow‐up care, particularly in guiding infant selection and counseling families. I would greatly welcome the authors' perspectives on this point and any additional data they may be able to share.

## Author Contributions

**Yudai Kaneda:** conceptualization, writing – original draft.

## Conflicts of Interest

The author declares no conflicts of interest.

## AI Use Statement

The author used ChatGPT (OpenAI, San Francisco, CA, USA) to assist with English language editing and translation of the manuscript. The tool was used solely for linguistic refinement. The author takes full responsibility for the content of the manuscript.

## Transparency Statement

Yudai Kaneda affirms that this manuscript is an honest, accurate, and transparent account of the research reported, that no important aspects of the research have been omitted, and that any differences from the planned research (and registration, if relevant) are explained by Yudai Kaneda.

## Data Availability

Data sharing not applicable to this article as no data sets were generated or analyzed during the current study.

## References

[1] A. Monfared , M. Amini , J. Abolghasemi , A. Mazouri , and Z. D. Boroujeni , “Effect of Massage With Verbal Contact by Mothers of Premature Infants on Maternal Attachment and Infant's Motor Performance: A Randomized Controlled Trial,” Health Science Reports 9, no. 2 (2026): e71697. 41641229 10.1002/hsr2.71697 PMC12865222

[2] A. A. Salas , W. A. Carlo , N. Ambalavanan , et al., “Gestational Age and Birthweight for Risk Assessment of Neurodevelopmental Impairment or Death in Extremely Preterm Infants,” Archives of Disease in Childhood ‐ Fetal and Neonatal Edition 101, no. 6 (2016): F494–F501. 26895876 10.1136/archdischild-2015-309670 PMC4991950

[3] Y. Kono , “Neurodevelopmental Outcomes of Very Low Birth Weight Infants in the Neonatal Research Network of Japan: Importance of Neonatal Intensive Care Unit Graduate Follow‐Up,” Clinical and Experimental Pediatrics 64, no. 7 (2021): 313–321. 33171036 10.3345/cep.2020.01312 PMC8255508

[4] E. H. Chung , J. Chou , and K. A. Brown , “Neurodevelopmental Outcomes of Preterm Infants: A Recent Literature Review,” Translational Pediatrics 9, no. Suppl 1 (2020): S3–S8. 32206579 10.21037/tp.2019.09.10 PMC7082240

[5] C. C. Guerra , M. C. de Moraes Barros , A. L. Goulart , L. V. Fernandes , B. I. Kopelman , and A. M. dos Santos , “Premature Infants With Birth Weights of 1500–1999 G Exhibit Considerable Delays in Several Developmental Areas,” Acta Paediatrica 103, no. 1 (2014): e1–e6. 24117765 10.1111/apa.12430

